# A Soluble Form of CTLA-4 Is Present in Paediatric Patients with Acute Lymphoblastic Leukaemia and Correlates with CD1d^+^ Expression

**DOI:** 10.1371/journal.pone.0044654

**Published:** 2012-09-25

**Authors:** Rita Simone, Claudya Tenca, Franco Fais, Matteo Luciani, Giulio De Rossi, Giampaola Pesce, Marcello Bagnasco, Daniele Saverino

**Affiliations:** 1 Department of Experimental Medicine – Section of Human Anatomy, University of Genova, Genova, Italy; 2 The Feinstein Institute for Medical Research, North Shore-LIJ Health System, Manhasset, New York, United States of America; 3 Department of Pediatric Haematology and Oncology, Ospedale Bambino Gesù, Rome, Italy; 4 Medical and Radiometabolic Therapy Unit, Department of Internal Medicine, University of Genova, Genova, Italy; University of Massachusetts Medical School, United States of America

## Abstract

CTLA-4 is a key factor in regulating and maintaining self tolerance, providing a negative signal to the T cell and thus limiting immune responses. Several polymorphisms within the *CTLA-4* gene have been associated with an increased risk of developing autoimmune diseases and, very recently, with susceptibility to human cancer. Acute lymphoblastic leukemia is a clonal disorder of lymphoid progenitors representing the most frequent malignancy of childhood. Here, we show the presence at significantly elevated levels of a circulating soluble form of CTLA-4 in 70% of B-ALL pediatric patients with active disease, the positive correlation between the percentage of leukemic B lymphocytes and the amount of serum sCTLA-4, and the expression of sCTLA-4 transcript by B cells in patients. Finally, a correlation between CD1d expression (a negative prognostic marker) and the sCTLA-4 in B-ALL patients was observed. This suggests a possible role of this soluble molecule as a marker of progression or severity of the neoplastic disease.

## Introduction

Acute lymphoblastic leukemia (ALL) is a clonal disorder of lymphoid progenitors with distinctive morphologic, immunophenotypic and genotypic features and represents the most frequent malignancy of childhood [1]. ALL affects T- or B-lineage precursor cells, the latter accounting for approximately 80% of the cases in Europe and the USA. Immunophenotype allows further subdivision of B lineage ALL into pro-B (CD19+, CD10−), common (CD19+, CD10+), pre-B (CD19+, cytoplasmic immunoglobulin (Ig)+) and mature B (surface Ig+). However, with some exceptions, this classification has little value for survival prediction [2]. Several prognostic factors that herald poor outcome have been described in childhood ALL [3], [4]. Infants less than 1-year of age (infant leukemia) have a poor rate of survival [1]. Thus, beyond a better understanding of the molecular mechanisms of disease and resistance to chemotherapy, the identification of new markers, potentially able to be used to guide the development of new targeted chemotherapies or immunotherapeutic agents, which may be used to intensify therapy in children with ALL who have poorer.

CD1d is a monomorphic molecule that provides a suitable target for immunotherapy in view of the characterization of a glycolipid, αλφα-galactosylceramide (αλφα-GalCer), capable of being presented to CD1d-restricted T cells with cytotoxic potential [5], [6]. We have previously demonstrated the expression of CD1d on leukemic B-cells in a proportion of high risk pediatric ALL patients with poor prognosis [7].

Cytotoxic T-lymphocyte-associated antigen-4 (CTLA-4) is a homodimeric glycoprotein belonging to the human Ig gene superfamily originally described on the surface of murine and human activated T cells [8]. The vast majority of in vitro and in vivo studies on CTLA-4 support its negative role on T-cell activation contributing to the physiologic termination of the immune response [13], [14] CTLA-4 inhibitory function occurs upon interaction with its ligands, CD80 (B7.1) and CD86 (B7.2), expressed on antigen-presenting cells (APCs), resulting in inhibition of IL-2, IFN-γαμμα, IL-4 cytokines production, IL-2 receptor expression and cell cycle progression [15], [16]. Several mechanisms of CTLA-4 function have been proposed including ligand competition with the positive T-cell costimulatory CD28 molecule [17], interference of TCR signalling [18] and inhibition of cyclin D3 and cyclin-dependent kinases (cdk4/cdk6) production [19]. A possible function of CTLA-4 in the regulatory role of suppressor CD4+CD25+ T cells has generated widespread interest indicating another mechanism by which CTLA-4 might downregulate immune responses [20] and also promote peripheral tolerance [21].

Otherwise, growing evidence supports its wider role as an immune attenuator which may also act in other cell lineages, such as B lymphocytes [9], monocytes and monocyte-derived dendritic cells [10]. In addition, the surface expression of CTLA-4 in neoplastic cell populations from different leukemia subtypes was demonstrated by Pistillo et al. [11]. These authors suggest a possibile physiopathological role of this receptor: the expression of CTLA-4 in leukemias (as well as in a number of human malignant solid tumors including carcinoma, melanoma, neuroblastoma, rhabdomyosarcoma and osteosarcoma) might indicate the ability to interact with the CD80/CD86 ligands on antigen-presenting cells, and to transduce a relevant immunosuppressive signal [11], [12].

A native soluble form of CTLA-4 (sCTLA-4), deriving from lack of transmembrane sequence, has been described [22]. High concentration of sCTLA-4 were observed in sera of patients with autoimmune thyroid diseases [22], [23], as well as in patients with other autoimmune diseases, such as type-1 diabetes [24], diffuse cutaneous systemic sclerosis [25], systemic lupus erythematosus [26], myasthenia gravis [27] and celiac disease [28]. In addition, raised plasma levels of sCTLA-4 were observed in patients with allergic asthma [29] and allergy to hymenoptera venom, but not in allergic rhinitis [30].

Soluble CTLA-4 may have important immunoregulatory functions. The effect of sCTLA-4 binding to CD80/CD86 molecules might depend on the activation state of the cells involved, interfering with T cell costimulation and with T cell responses. Thus, sCTLA4 might act indirectly both as inhibitor or as enhancer of the immune response [22], [29], [31].

In addition, elevated levels of soluble CD80 [32], [33], and CD86 in some leukemia patients have been demonstrated, and elevated sCD86 levels have been reported as a marker of poor prognosis in acute myeloid leukemia [34], [35].

However, the mechanisms for the production of sCTLA-4, sCD28, sCD80 and sCD86, and their correlation with hematological malignancy activity have not been well elucidated.

As previously mentioned, leukemic B-ALL cells have been proved to be able to express CTLA-4 on their surface [11]. Pistillo et al. evidentiated an apparent different expression of CTLA-4 on the membrane of leukemic B cells depending on the mAb utilized. Flow cytometric analysis carried out with a panel of anti–CTLA-4 human single chain fragment of variable domain antibodies, all containing leukemic cells at high frequency (greater than 75%) in peripheral blood or bone marrow aspirate samples. On the other hand, when commercially available mAb were used, the percentage of CTLA-4 expression on leukemic B cell surface decrease to 20% (vs. 90% in cytoplasm) [11].

The release of a soluble form of CTLA-4 provides a potentially powerful means by which APC and/or malignant cells may modulate the co-stimulatory signals normally delivered by the membrane form of CTLA-4. In an attempt to further evaluate the immunopathological roles of T-cell costimulatory molecules and to search for potential surrogate markers in ALL, we investigated the plasma concentration of sCTLA-4 in ALL pediatric patients, compared with normal healthy subjects and their relationship with specific membrane-expressed markers.

## Materials and Methods

### Patients

The study population included 80 children with ALL of B-cell precursor origin. ALL cases were part of a bank of cryo-preserved samples collected between 1995 and 2008. Samples were analyzed on the basis of the availability of frozen sera and, therefore, are not representative of a consecutive series of patients. Clinical data included age, sex, white blood cell count at diagnosis, assessment of hepatomegaly, splenomegaly, and BM and central nervous system (CNS) involvement [7]. Written informed consent was obtained from parents, all samples were obtained following the ethical guidelines of the most recent Declaration of Helsinki (Edinburg 2000) and approved by the Ethics Committee of Ospedale Bambino Gesù, Italy. Diagnosis and categorization resulted from morphologic analysis of BM aspirates, immunophenotyping, and cytogenetic and molecular genetic analysis of PB and/or BM samples [7]. Immunophenotyping included analysis with a standard panel of monoclonal antibodies (mAb) to CD19, CD10, CD34, cytoplasmic and surface Ig. Cases were classified as pro-B ALL (n = 8), common ALL (n = 54), pre-B ALL (n = 18). No mature B-ALL cases were observed in this series. Classification of ALL according to age allowed identification of six infant ALL (<1 year) and 74 non-infant ALL cases.

Fourty-five age matched normal serum samples were obtained (age range from 2 to 6), after parents’ written informed consent, from children undergoing diagnostic laboratory procedures, which did not reveal hematological abnormalities neither autoimmune disorders. In addition, it is well known that sCTLA-4 levels are virtually undetectable in normal controls [22], [23], [28]–[30]. Otherwise, this report shows for the first a description of normal values in a pediatric group.

### Antibodies and Immunostaining

For ALL cell staining the following mAb were used: CD1d-phycoerythrin (PE), CD10-PE, CD10-fluorescein isothiocyanate (FITC), CD38-FITC, CD19-peridinin chlorophyll protein-cyanine dye 5.5. (PerCP-Cy5.5), CD34-FITC, CD3-FITC, CD19-FITC, CD80-FITC, CD86-FITC, CD11a-FITC and fluorochrome-conjugated isotype-matched control mAbs (Becton Dickinson Italia, Milan, Italy). B-CLL cells (2×10^5^) were incubated for 20 min with mAb at 4°C. Cells were then washed and resuspended in 300 µιχρο liters cold PBS before flow cytometric analysis (FACScalibur, Becton Dickinson), using the CellQuest software (Becton Dickinson).

### ELISA

Specific ELISA kits were used for measuring serum sCTLA-4 levels (Bender), according to the manufacturer’s protocol. Each sample was diluted 1∶10 and tested in triplicate. Deviation between triplicates was <10% for any reported value. The lowest sensitivity threshold is 0.1 pg/ml.

The analytical response was linear approximately between 0.162 and 1.200 of absorbance values (corresponding to 0.1–50 ng/ml) as assessed by serial dilution test using a strongly positive serum (data not shown).

#### Western blotting

Western blotting was used to detect serum sCTLA-4. Proteins were separated by 10–20% gradient PAGE in a discontinuous buffer system on a Mini-Protean system (Bio-Rad, Segrate, Milano, Italy). The separated components were electroblotted onto PVDF membranes. The blots were washed with 0.15 M NaCl, 0.05 M Tris (TBS), pH 7.5, with 0.3% Tween 20 and reacted with a 1∶100 dilution of the anti-CTLA-4 mAb (clone 14D3, IgG2a, eBiosciences, San Diego, CA, USA) for 1 h at room temperature, washed, and then reacted with reporter antibody (HRP-conjugated anti-mouse IgG) for 1 h. The blots were then developed by the use of a commercially available chemiluminesence detection kit (BMB, Indianapolis, IN) according to the manufacturers instructions.

#### mRNA analysis by RT-PCR

B lymphocytes were separated from peripheral blood mononuclear cells, and purified *in vitro* using the MACS CD20 isolation kit (Miltenyi Biotech, Auburn, CA USA). Total RNA was extracted using RNAse Mini kit (Qiagen, Milano, Italy) according to the manufacturer’s recommendations. The single-stranded cDNA was synthesized using 1 µg of total RNA by reverse transcription by incubating it at 42°C for 1 h with 20 pmol of oligo(dT) (Roche Molecular Biochemicals, Basel, Switzerland), 500 µM dNTPs (Roche Molecular Biochemicals), 30 U of RNase inhibitor (5 Prime 3 Prime, Boulder, CO), and 200 U Moloney murine leukemia virus reverse transcriptase (Life Technologies, Gaithersburg, MD) in a total volume of 20 µicroL.

PCR reactions were performed with cDNA and primers designed to amplify the entire coding sequence of CTLA-4∶5′-ATGGCTTGCCTTGGATTTCAGCGGCACAAGG-3′ and 5′-TCAATTGATGGGAATAAAATAAGGCTGAAATTGC-3′ (predicted size: 672 bp). PCR reaction was as follows: 94°C for 5 min, 30 cycles 94°C for 1 min, 60°C for 1 min and 72°C for 1 min followed by a final extension at 72°C for 5 min. The amplified fragments were separated on 1% agarose gel and visualized by ethidium bromide. As controls, the G3PDH primers were used: 5′-ACATCGCTCAGAACACCTATGG-3′, and 5′-GGGTCTACATGGCAACTGTGAG-3′.

### Statistical Analysis

Statistical analysis was performed using GraphPad Prism software 4·0 (GraphPad Software Inc., CA, USA). Because of the skewed nature of some of the data non-parametric tests were used throughout. Associations were calculated using Spearman rank correlations. Differences between unpaired groups were evaluated by the Mann–Whitney *U*-test. *P* values *<*0.05 were considered significant.

## Results

### Plasma sCTLA-4 Levels in Patients with ALL

**Figure 1:**
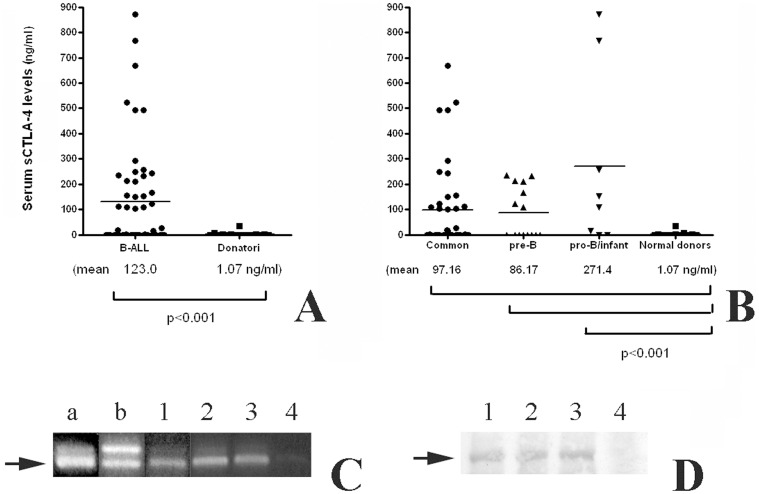
A sCTLA-4 is found in serum of B-ALL patients. *Panel A*, The concentration of sCTLA-4 was evaluated by ELISA on sera collected from B-ALL patients (n = 64), and a healthy donor group as a control (n = 45). Results are expressed as nanogram per milliliter. Each sample was diluted 1∶10 and tested in triplicate. Samples higher to the detection limit of the test (50 ng/ml) were diluted appropriately and tested again. Deviation between triplicates was <10% for any reported value. Arrow marks 23 kDa species. *Panel B*, The ALL patients were classified in relation to the membrane immunophenotype of peripheral blood mononuclear cells (PBMCs) in three groups: pro-B ALL (n = 8), common ALL (n = 41), pre-B ALL (n = 15). The analysis of sera sCTLA-4 showed a statistically difference between the three ALL groups and the control samples. No significant differences were observed among the three ALL groups. *Panel C*, Reverse transcription–PCR was performed in order to verify the presence of the spliced variant of CTLA-4 on PBMCs from a healthy donor (*a*), on freshly isolated T lymphocytes (*b*), and from three representative B-ALL patients (*1* to *4*; sera of patients from *1* to *3* resulted positive for sCTLA-4 ELISA, *4* was negative). The amplification by PCR of the human CTLA-4 coding sequence revealed the constitutive expression by PBMCs from a normal donor of two transcripts whose sizes are: 650 and 550 bp. Otherwise, B-ALL patients preferentially express the light transcript. Arrow marks a 550 bp sCTLA-4 fragment. *Panel D*, Immunoprecipitation of sCTLA-4 was performed on sera from a representative group of B-ALL patients. *1*, *2* and *3* sera were resulted positive during ELISA analysis (showing a mRNA band of 550 bp); *4* was negative (these results were in line with those obtained by PCR).

In this study, the presence of circulating sCTLA-4 was evaluated in the sera of all normal donors and patients enrolled in this study (Fig. 1, panel A). Plasma sCTLA-4 levels in 54 normal blood donors ranged from 0.00 ng/ml to 36.06 ng/ml (mean ± standard deviation, 1.071 ng/ml ±5.318 ng/ml). A wider range of levels was observed in paediatric patients with ALL. Among patients with ALL, sCTLA-4 levels ranged from 0.02 ng/ml to 870.8 ng/ml (132.0 ng/ml ±208.7 ng/ml). The majority of patients with B-ALL (42%) had sCTLA-4 levels higher the range observed in normal donors, and, overall, there was a significant difference between the levels detected in normal donors and the levels in either patients with B-ALL (*P*<0.0001).

In addition, we confirmed the presence of sCTLA-4 mRNA in PBMCs isolated from ALL patients. As shown in Fig. 1, panel C, a mRNA form encoding for sCTLA-4 was evident, suggesting that the molecule we measured in ELISA tests was a result of an alternative splicing.

Finally, Western-blot of human serum was probed with specific mAb anti-CTLA-4. As shown in Fig. 1, panel D, three different samples resulted positive by ELISA analyses (BBG14, BBG33 and BBG56), were characterized by a band of approximately 23 kDa, which is consistent with previous reports [23], [29], [31].

### Association of sCTLA-4 Levels with Immunophenotype Classification

The ALL patients were classified in relation to the membrane and cytoplasmic_immunophenotype of peripheral blood mononuclear cells (PBMCs) in three groups: pro-B ALL (n = 8), common ALL (n = 41), pre-B ALL (n = 15). The analysis of sera sCTLA-4 showed a statistical difference between the three ALL groups and the control samples (Fig. 1, panel B). The results obtained evaluating serum sCTLA-4 concentrations in the 3 subgroups demonstrated a different ability of the 3 groups in producing sCTLA-4. As shown in Fig. 1B, serum levels of sCTLA-4 ranged from 0.0 ng/ml to 669.7 ng/ml (97.16±169.3 ng/ml) in the common ALL group, from 0.0 to 236.0 (86.17±101.1 ng/ml) in pre-B ALL, and from 0.0 to 870.8 (271.4±350.3 ng/ml) in pro-B ALL. As resulted from statistical analyses, no correlation was evidenced among the three ALL groups. Otherwise, all of the three groups of patients were significantly different from the donor group (*p*<0.005).

### Association of Serum sCTLA-4 Levels with HLA-DR, CD19, CD10, CD3, and CD38 Expression on PBMCs in ALL Patients

**Figure 2:**
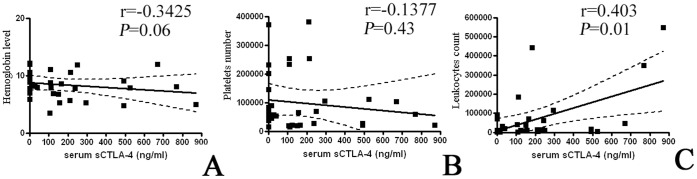
Analyses of correlation between sCTLA-4 and some clinical characteristics in B-ALL patients. As specified in the text, the only significant correlation was between the sCTLA-4 levels and the leukocyte counts (*Panel C*). No significant correlation was observed between sCTLA-4 levels and either hemoglobin levels (*Panel A*), or platelet numbers (*Panel B*).

There was no significant correlation between sCTLA-4 levels and either age (data no shown), hemoglobin levels, or platelet numbers in either patient group (Fig. 2, panels A and B). However, the levels of sCTLA-4 in patients with ALL were correlated with leukocyte counts (Fig. 2, panel C).

**Figure 3:**
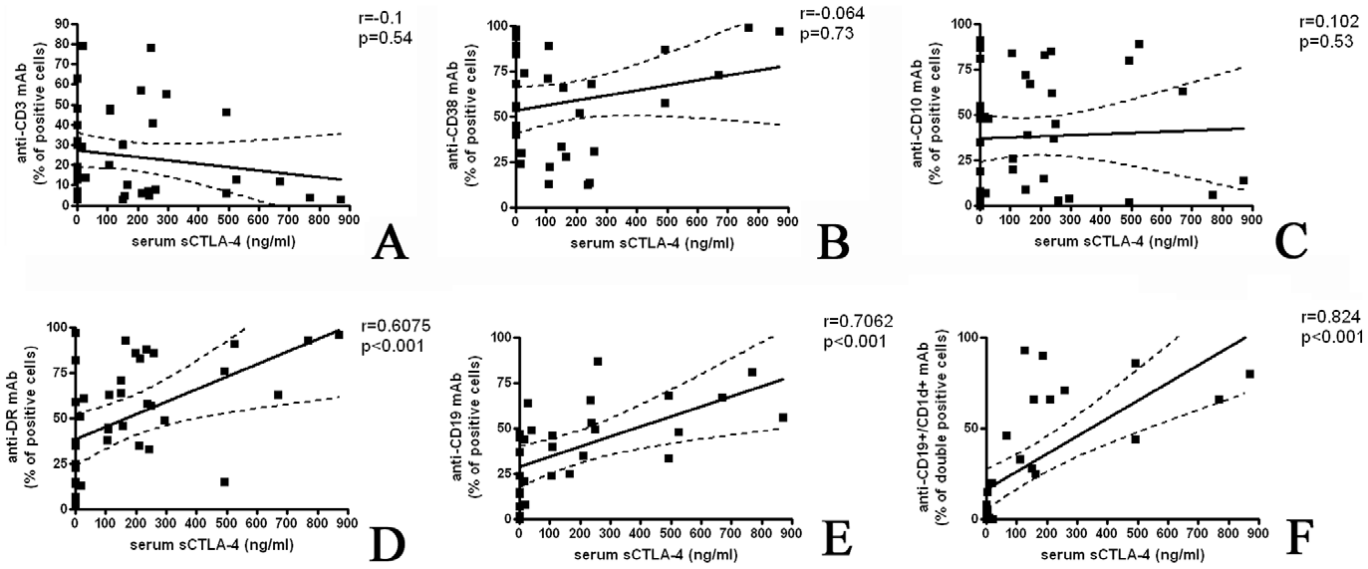
A correlation between sCTLA-4 and neoplastic B cells is apparent. The surface immunophenotype of PBMCs from B-ALL were stained with different mAb and the percentage of positive cells was compared with the level of sCTLA-4 measured in the corresponding serum. The correlation between serum sCTLA-4 levels and the percentage of CD3 expressing cells (T lymphocytes), as well the correlation with CD38 (early and late stages of the B and T-cell maturation) and CD10 expression (a diagnostic marker of human acute lymphocytic leukemia) were no significant (*Panels A, B* and *C*). Otherwise, there was a significantly positive correlation between the level of sCTLA-4 and the frequency of HLA-DR and CD19 membrane expressing cells (*Panels D* and *E*). In addition, a positive correlation between the sCTLA-4 levels and the percentage of CD19^+^/CD1d^+^ cells (marker correlated with poor prognosis) was observed (*Panel F*).

PBMCs of patients with ALL were stained with different mAb and the percentage of positive cells was compared with the level of sCTLA-4 measured in the corresponding serum. Surprisingly, the correlation between serum sCTLA-4 levels and the percentage of CD3 expressing cells was no significant (*r* = 0.1, *p = *0.54) (Fig. 3, panel A). In fact it is described that T lymphocytes are able to produce the spliced soluble form of CTLA-4. On the other hand, there was no significant correlation also when CD38 expression (a receptor preferentially expressed at both early and late stages of the B and T-cell maturation) and CD10 expression (one of the most significant cell surface marker in the diagnostic of human acute lymphocytic leukemia) were compared to the level of sCTLA-4 (*r* = 0.0647, *p = *0.73, and *r* = 0.1022, *p = *0.53 respectively) (Fig. 3, panels B and C respectively). Otherwise, there was a significantly positive correlation between the level of sCTLA-4 and the frequency of HLA-DR and CD19 membrane expressing cells (*r* = 0.6075, *p*<0.001 and *r* = 0.7062 *p*<0.001, respectively) (Fig. 3, panels D and E).

### Plasma sCTLA-4 Levels and CD1d

We have previously observed that CD1d is expressed in ALL subsets with poor prognosis and that CD1d^+^ blasts are able to interact with NKT cells via CD1d [7]. For these reasons we analyzed the correlation between these markers. The amount of sCTLA-4 levels in patients with ALL were compared to the percentage of CD19^+^/CD1d^+^ cells. As shown in Fig. 3 panel F, there is a positive correlation between these two markers (*r* = 0.824, *p*<0.001).

## Discussion

*CTLA-4* was first described as an important negative regulator of T-cell activation and proliferation, interacting with B7 molecules on antigen-presenting cells. It is therefore a plausible candidate as a susceptibility gene in diseases with T-cell mediated pathogenesis. Expression and function of CTLA-4 inhibitory receptor have been investigated mainly in T lymphocytes [13]–[17]. More recently, the expression of membrane CTLA-4 is demonstrated also on cells different from T lymphocytes, such as B cells, monocytes or dendritic cells [9], [10] in correlation with the activation state of the cell. Of note, the expression of CTLA-4 at the cellular membrane level is often controversial, mainly due to the low levels of expression combined with poor capability of commercial mAb anti-CTLA-4. Otherwise, the expression of this inhibitory receptor was previously demonstrated not only on the membrane of T and B lymphocytes, but also on different tumor cells [8]–[12].

In addition, alternative splicing of mRNA encoding the CTLA-4 receptor leads to the production of a molecule (sCTLA-4) that lacks a membrane anchor and is therefore secreted into the extracellular space. There is abundance of literature showing the presence of sCTLA-4 in autoimmune diseases [20]–[28], and also in allergic asthma [29] and allergy to hymenoptera venom [30].

The release of sCTLA-4 from acute lymphoblastic leukaemia cells may constitute a strategy for immune-surveillance escape. In fact, the effect of sCTLA-4 binding to CD80/CD86 molecules might depend on the activation state of the cells involved, interfering with T cell costimulation and with T cell responses. Thus, sCTLA4 might act indirectly both as inhibitor or as enhancer of the immune response [23], [28], [30], [31].

Association of type-1 diabetes, Graves’ disease and CD with a point mutation in exon 1 of CTLA-4 (*i.e.* position 49A/G) leading to a Thr/Ala substitution in the leader peptide has been described in several different populations [16]–[22]. Moreover, single nucleotide polymorphisms (SNPs) with a point mutation in exon 1 of *CTLA-4* gene have been linked to susceptibility to several autoimmune disease [31]. In fact, higher levels of sCTLA-4 were observed in individuals with +49A/G and CT60A haplotype. Interestingly, although the data in this field of research remain controversial, it has been recently shown that the same SNPs in the CTLA-4 coding region are also associated with susceptibility to human cancer [36], such as cervical squamous cell carcinoma [37], colorectal cancer [38], hepatocellular carcinoma [39], gastric cancer [40] and acute myeloid leukemia [41]. These results suggest that expression of CTLA-4 would be associated with a decreased ability of the immune system to detect and eliminate tumor-associated antigens. Thus, the presence of a more tolerogenic CTLA-4 genotype, for instance related to a more frequent production of sCTLA-4, could represent a potential form of tumor immune evasion.

In the present study we demonstrate the presence at significantly elevated levels of a circulating soluble form of CTLA-4 in 70% of B-ALL pediatric patients with active disease. In these series of experiments, data of expression of surface CTLA-4 are not available to us, however we presume that it could be similar to previous results published [11], [12]: neoplastic cells from most B-CLLs were positive for all CTLA-4 epitopes at either the surface or the cytoplasm.

The elevation of SCTLA4 observed in this series of patients appear to somehow conflict, by a theoretical point of view, with the observation of increased sCTLA-4 serum levels in patients with autoimmune diseases. However, it should be considered that, at least in an in vitro system, sCTLA-4 is able to suppress proliferation of T lympocytes (both in a MLR and in an antigen specific experimental conditions] [23], [30].

Finally, it is known that B lymphocytes are able to produce CTLA-4 [11]. In this report, it is shown that sCTLA-4 transcript is expressed also by malignant B cells, at least in ALL paediatric patients.

The correlation observed among CD1d expression [7] and the higher levels of sCTLA-4 in B-ALL patients suggest a possible role of this soluble molecule as a marker of progression to malignancy, or as a marker of severity of the neoplastic disease. Unfortunately, we cannot yet definitely support this hypothesis. It should be interesting to follow-up the serum expression of this molecule and, possibly, to correlate its levels to the decreasing of neoplastic cell numbers following therapy.
